# Gender differences in reward and punishment for monetary and social feedback in children: An ERP study

**DOI:** 10.1371/journal.pone.0174100

**Published:** 2017-03-27

**Authors:** Ying Ding, Encong Wang, Yuchen Zou, Yan Song, Xue Xiao, Wanyi Huang, Yanfang Li

**Affiliations:** 1 State Key Laboratory of Cognitive Neuroscience and Learning, Beijing Normal University, Beijing, China; 2 Collaborative Innovation Center of Assessment toward Basic Education Quality, Beijing Normal University, Beijing, China; 3 Brain and Cognition Laboratory, Department of Psychology, Sun Yat-Sen University, Guangzhou, China; University of Pennsylvania, UNITED STATES

## Abstract

Gender differences in feedback processing have been observed among adolescents and adults through event-related potentials. However, information on whether and how this feedback processing is affected by feedback valence, feedback type, and individual sensitivity in reward/punishment among children remains minimal. In this study, we used a guessing game task coupled with electroencephalography to investigate gender differences in feedback processing, in which feedback to reward and punishment was presented in the context of monetary and social conditions. Results showed that boys were less likely to switch their response after punishment, had generally less feedback-related negativity (FRN) amplitude, and longer FRN latency in monetary and punishment conditions than girls. Moreover, FRN for monetary punishment, which is related to individual difference in reward sensitivity, was observed only in girls. The study provides gender-specific evidence for the neural processing of feedback, which may offer educational guidance for appropriate feedback for girls and boys.

## 1. Introduction

The appropriate processing of external feedback and changing behavior is indispensable for optimizing learning and goal-directed behavior [1]. The cognitive process of monitoring external feedback is called “feedback processing” [2]. The development of feedback processing is particularly important during childhood given that children are constantly facing new and frequently challenging learning experiences in social and educational settings [3]. Moreover, studies have found that the neurophysiological mechanisms of several cognitive skills are differentiated between boys and girls during childhood [4]. These skills include error processing, which reflects the same underlying reinforcement learning process as feedback processing [5]. Behavioral studies have also found gender differences in feedback processing during childhood [6, 7]. However, relatively few studies have been conducted on gender differences in the electrophysiological response to performance feedback during childhood.

### 1.1. Interpretation of Feedback-Related Negativity (FRN)

The neurophysiologic mechanisms that underlie the evaluation of external feedback are reflected by the components of event-related potentials (ERPs), which are identified as feedback-related negativity (FRN). FRN is a negative deflection that peaks at approximately 200–300 ms following the onset of the presentation of feedback [8, 9]. Recent studies have identified FRN sources in the anterior cingulate cortex (ACC) [10, 11], medial prefrontal cortex [11, 12, 13], and ventral striatum [11, 12, 14, 15]. Two main hypotheses are used to interpret FRN [16]. In the first hypothesis, FRN reflects a quantitative prediction error [17]. In the second hypothesis, FRN reflects conflict-related activities to fulfill the requirements for behavioral adjustments [16]. One prominent aspect of feedback is valence, which indicates whether the outcome of an action is positive or negative [16]. Miltner et al. [9] first found that FRN was elicited by negative outcomes. Subsequent studies have shown that FRN can be observed following both rewards and punishments, particularly if they are unexpected [3, 18]. Type is another prominent aspect of feedback. Previous electrophysiological studies frequently used monetary feedback. In real life, however, monetary feedback is not the only feedback type that influences subsequent behavior. Social feedback, such as smiling, is also a strong reinforcement for children’s learning [19]. However, minimal information is available regarding how social feedback differs from other more concrete feedback, such as money. To our knowledge, only one study has used ERPs to measure the delivery phase of feedback processing in the context of monetary and social conditions among adults. Flores et al. [20] found that reward feedback elicited more negative FRN than nonreward feedback in the case of monetary feedback among adults, whereas the opposite result was obtained in the case of social feedback during the delivery phase of feedback processing. These researchers suggested that the neural activity involved in feedback processing can be specific to a feedback type among adults. However, whether the different neural activities observed in adults for monetary and social feedback also occur in children remains unclear. Therefore, in the present study, we focus on the neural mechanisms for monetary and social feedback during childhood.

### 1.2. Gender differences in feedback processing during childhood

Evidence from neuroimaging studies has indicated that gender differences occur during the brain development of neural systems associated with feedback processing in childhood. For example, Wood et al. [21] found that girls had slightly smaller straight gyrus volume than boys. Moreover, a small straight gyrus volume significantly correlated with good social perception only in girls. These findings suggested that the robust neural circuitry that supports social perception in females diverges from that in males beginning in childhood. Alarcó et al. [22] found that girls exhibited more integration of superficial amygdala (socio-affective processing) and parieto-occipital cortex than boys during at the ages of 10–16 years. All the aforementioned fMRI studies suggest that gender difference exists in socio-affective processing.

Although recent studies have reported gender differences in feedback processing as assessed electrophysiologically during childhood, these studies have mostly focused on monetary feedback, and their results are conflicting. Kujawa et al. [23] investigated neural reactivity to monetary rewards and losses at the age of 9 years. They found that boys had a larger ΔFN (losses minus gains) than girls. By contrast, Bress et al. [24] found that ΔFN did not differ between boys and girls at the ages of 8–13 years. The investigation of gender differences in feedback processing is not the main objective of these two studies; hence, one potential issue in these works is that the proportion of boys and girls are not matched. Crowley et al. [25] adopted a nonlearning reward versus nonreward task to compare feedback processing in children. They found that gender differences do not only exist in the amplitude of FRN, but also in its latency.

Moreover, how neural responses to social feedback and more concrete feedback, such as money, differ between girls and boys during childhood remains unclear. To address these issues, we examined gender as an explanatory factor in this investigation and focused on processing both monetary and social feedback during childhood by using an electrophysiological method.

### 1.3. FRN as a neural correlation of sensitivity to reward and punishment

Individuals with different sensitivities to reward and punishment can have varying affective reactions and motivational states to feedback. An increasing number of studies have examined the relationship between individual sensitivity to reward and punishment and ERP response to reward and punishment in FRN in adolescents and adults [3, 24]. For example, de Pascalis et al. [18] found that higher sensitivity to punishment was related to longer FRN latency. Their findings suggest that individual sensitivity to reward and punishment may affect the time course of feedback processing for reward and punishment, which may reflect as FRN latency. However, the relationship between FRN and individual sensitivity to reward and punishment during childhood remains unclear. In addition, a prior study found that gender differences existed in the progressive development of the anterior P300 at ages 6–17 years. In particular, anterior P300 was more prominent and bilateral in girls than in boys. This research also found that the right frontal P300 amplitude was related to reaction time only in girls [26]. Such findings possibly have gender-specific origin mechanisms that underpin the ERP component. Thus, clarifying the role of gender in the relationship between the neural activity of feedback processing and sensitivity to reward/punishment among typically developing children is significant.

### 1.4. Present study

In summary, the primary objective of the current study was to investigate gender differences in the neural processing of monetary and social reward and punishment feedback during childhood. We adopted a guessing game task to avoid contaminating the learning effects of our results [19]. As pointed out by Kohls and Herpertz- Dahlmann [27], both the interest in and understanding of the concept of money considerably increases between the ages 5 and 7 years among children, and are fully established by age 8 [28, 29]. Therefore, to avoid misunderstanding the concept of money, we select children ages 9 to 12 years (i.e., later childhood) to participate in our study. We hypothesized that FRN in the context of monetary and social feedback was affected by gender based on gender differences in the behavioral level of feedback processing and in the brain areas associated with feedback processing [21, 30]. The second objective of the current research was to examine the relationship between FRN and individual differences in reward and punishment for both boys and girls. We hypothesized that FRN could be significantly predicted based on sensitivity to both reward and punishment, and the prediction pattern would be influenced by gender. With regard to the direction of gender differences, we did not formulate any hypothesis beforehand.

## 2. Methods

### 2.1. Participants

A total of 56 children (29 boys and 27 girls, ranging in age from 9 to 12 years) were recruited through online advertisements for this study. Data from 8 participants (3 boys and 5 girls) were discarded because of the high noise ratio in the electroencephalography (EEG) signals. Data from 4 other participants (2 boys and 2 girls) were also discarded because of excessive horizontal and vertical eye movements. Therefore, 44 children (24 boys and 20 girls) were used in the final analyses. The mean age of the children was 9.84 years (SD = 1.07). No significant gender difference in age was determined (mean age: 9.79 (boys) versus 9.90 (girls), *t*(42) = −0.24, *p* > 0.05). All the children completed the Raven Standard Progressive Matrices (SPM) [31], which was used to measure their nonverbal intelligence [32]. Intellectually, the participants were within the normal range (Raven SPM raw scores, mean = 49.66, SD = 5.28). No gender difference was observed in the Raven SPM raw scores (Raven SPM raw scores mean: 50.96 (boys) versus 48.35 (girls), *t*(42) = 1.63, *p* > 0.05). The current research was approved by the Beijing Normal University Institutional Review Board, and written informed consent was obtained from the primary caregiver (typically the mother) of each participant.

### 2.2. Behavioral testing

The assessments of reward sensitivity and punishment sensitivity were evaluated using the Sensitivity to Punishment and Sensitivity to Reward Questionnaire for children (SPSRQ-C), which was a caregiver report measure [33]. The SPSRQ-C contained 33 items, which included a Punishment Sensitivity subscale (SP; 15 items) and a Reward Sensitivity subscale (SR; 18 items) [34]. Each item was scored on a 5-point Likert scale (1 = never, 2 = seldom, 3 = sometimes, 4 = often, 5 = always). The internal consistency in the present sample is good (SP: Cronbach’s α = 0.879, SR: α = 0.861).

### 2.3. Experimental stimuli and task

**Fig 1:**
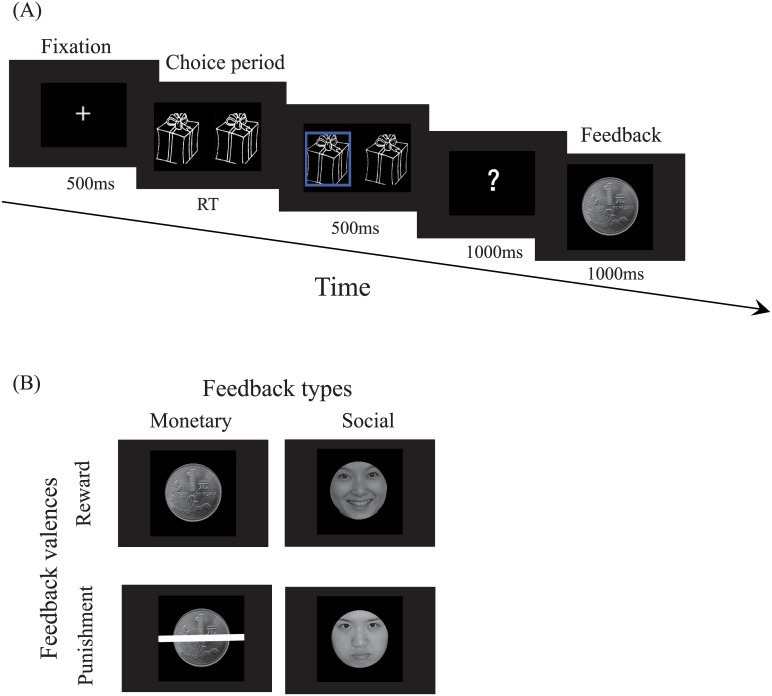
Stimulus presentation. (A) Illustration of the paradigm. (B) Illustration of the monetary (left) and social (right) feedback conditions. Reward feedback is shown on top, and punishment feedback is shown below.

A simple guessing task, which was adopted from Stavropoulos and Carver [19], was used in this study. Each trial started with the presentation of a fixation cross (1.44° × 1.44°) at the center of the screen for 500 ms, followed by images of two lifelike gift boxes along a row centered on the screen (16.77° × 7.16°); one of these boxes had a gift inside. The participants were instructed to choose one box which they believed had a gift inside it by clicking on the “F” key on the keyboard using their left forefinger for the left box or the “J” key on the keyboard using their right forefinger for the right box. The images of the boxes remained on the screen until the participant made a choice. Then, the chosen box was highlighted with a thick blue border for 500 ms to reinforce the idea that the participants had control over the task and their responses were being recorded [19]. Then, a question mark (?) would appear at the center of the screen for 1000 ms, covering an area of 1.8° × 3.38° of the visual angle. Finally, feedback was presented on the screen for 1000 ms (Fig 1A).

Blocked feedback has two types: monetary and social. The incidental stimulus for the monetary feedback type was a picture of a ¥1 coin (8.58° × 8.58°) gained or lost. The incidental stimulus for the social feedback type was a picture of a smiling or frowning face (8.58° × 8.58°) from the native Chinese Facial Affective Picture System [35, 36]. The participants were told that if they responded correctly, a ¥1 coin or a smiling face would be used as the reward feedback. By contrast, a ¥1 coin was crossed off with a white line (13.54° × 1.07°) or a frowning face was used as the punishment feedback (Fig 1B). To ensure that less than three of the same feedback would appear in a row, a computer program was adopted to predetermine reward versus punishment feedback in pseudorandom order.

The guessing task consisted of 8 blocks. Each block had 60 trials that consistently offered either monetary or social feedback in a pseudorandom order. In each block, feedback was rigged to have the probability of 50% reward and 50% punishment outcome. E-Prime software was used to present all stimuli against a black background on a 21-inch cathode ray tube (CRT) monitor, with a viewing distance of approximately 80 cm. Although feedback was also randomized, i.e., no pattern of a certain rule predicted specific outcomes, the children were led to believe that some people “could sometimes figure out a pattern” [25].

### 2.4. Procedure

First, the participants completed the Raven SPM, while their caregivers were required to finish SPSRQ-C. Before EEG recording, each child was first shown 10 potential gifts and was asked to order them by selecting the best, second best, and third best gifts. Then, the participants were seated comfortably in a dimly lit, sound-attenuating, and electromagnetically shielded room. The EEG electrodes were placed, and a brief instruction was given about the task. Furthermore, the participants were provided with five trials to practice. Subsequently, the participants started the experiment by reading the experimental instruction that appeared on the screen. Then, they were told to press any button to begin as soon as they were ready. During the experiment, the participants were required to observe the stimuli presented on the screen and to remain still. Each block lasted 2–3 min. The participants could rest briefly between blocks to reduce fatigue. Similar to a previous study, the participants were compensated for their time at the end of the experiment and received their selected gift bought using the money earned during the guessing game with the monetary feedback type; no additional gift was received for the guessing game with the social feedback type [20]. Moreover, to increase ecological validity, we have followed the study of Mushtaq et al. [37] and told the participants that they would be remunerated based on their performance. However, all the subjects received the same amount of money due to the random nature of the results. In the present study, we have replaced money with the best gift appropriate for each child.

### 2.5. Electrophysiological recording and data reduction

Continuous EEG recording were collected using an elastic cap and the 128 channel system (HydroCel Geodesic Sensor Net, Electrical Geodesics, Inc., Eugene, OR, USA) with Net Station EEG Software. The impedance of all of the electrodes was maintained below 50 kΩ during data acquisition. All the electrodes were physically referenced to Cz (fixed by the EGI system). EEG was amplified with a band pass of 0.01–100 Hz and digitized online at a sampling rate of 500 Hz.

Off-line EEG processing and analyses were performed by adopting custom MATLAB (MathWorks) scripts using functions from the EEGLAB environment [38]. The EEG data were first band-pass filtered (1–40 Hz) and then re-referenced to the average of two electrodes on the left and right mastoids. In the next step, data were inspected for noisy electrodes, which were excluded from further analysis. Data were subsequently inspected for gross movements and muscle artifacts. The trimmed data were then decomposed into maximally independent component processes using temporal independent component analysis (ICA) decomposition via extended infomax ICA components associated with horizontal and vertical eye movements that were visually identified and removed according to their spatial, spectral, and temporal properties. EEG was then segmented relative to the feedback onset (−200 ms to 800 ms), and a 200 ms window from −200 ms to 0 ms prior to feedback onset served as the baseline. After artifact rejection, the average number of valid trials for monetary reward was 84.82 (SD = 19.42). The valid trials for monetary punishment was 84.68 (SD = 20.05), the valid trials for social reward was 80.0 (SD = 20.56), and the valid trials for social punishment was 84.30 (SD = 20.84). The number of trials did not differ between monetary and social conditions (*p* > 0.05). No significant difference between gender groups was observed for the average number of valid trials under each condition (*F*(1, 42) = 2.35, *p* > 0.05). Lastly, separate averages were calculated for each combination of feedback valence (reward versus punishment), feedback type (monetary versus social), and gender group (boys versus girls), which resulted in eight average waveforms for each electrode.

### 2.6. Data analysis

#### 2.6.1. Behavioral measures

Behavioral performances were analyzed in terms of sensitivity to reward and punishment, mean reaction time, and switching percentages. For sensitivity to reward and punishment, an independent sample *t*-test was analyzed. For mean reaction times and switching percentages, which were computed as functions of the outcome on the preceding trial [16], repeated-measure ANOVA were performed using the following design: the within-subject factor feedback valence (reward versus punishment) and feedback type (monetary versus social), and the between-subject factor gender group (boys versus girls).

#### 2.6.2. ERPs

**Fig 2:**
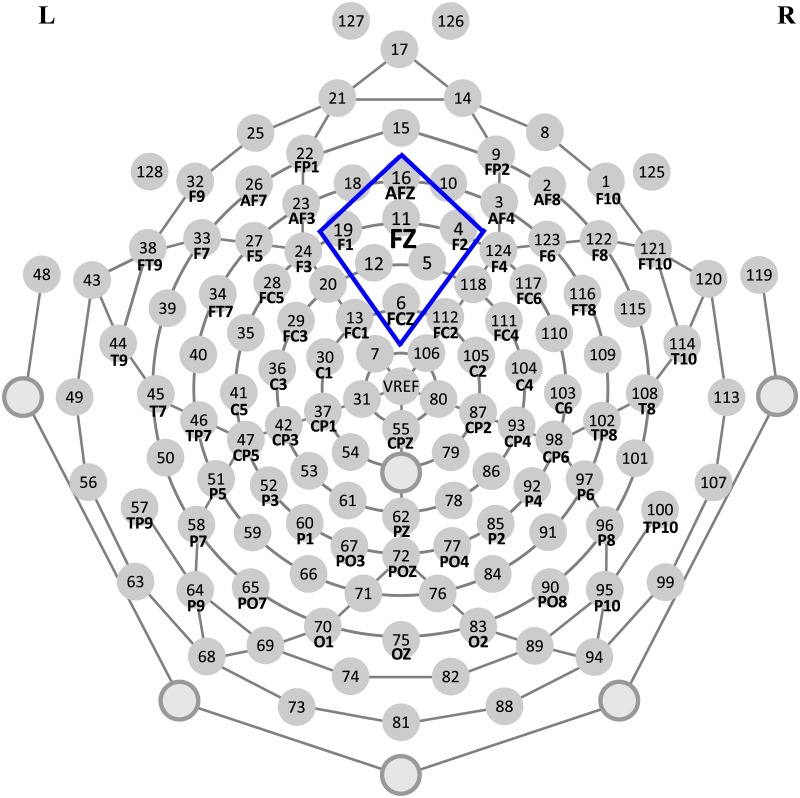
Selected medial frontal channels that surround Fz from a 128 electrode dense array (Electrical Geodesics, Inc.).

As mentioned in the Introduction section, FRN is an ERP component that is associated with feedback processing. Therefore, we focused on the mean peak amplitude and latency of FRN in this study. Previous studies have suggested that FRN would be localized to the midline frontal region along the midline at site Fz [25]. We relied on the average signal of seven electrodes positioned around Fz, particularly electrode numbers Afz, Fz, FCz, F1, F2, 12, and 5 (Fig 2). The amplitude of FRN was calculated as the mean value of a 20 ms window centered at the minimum value of the grand average waveforms between 200 ms and 300 ms within the electrode cluster. To determine raw ERP component latency, the current study identified the latency associated with the peak amplitudes of FRN [39] within 200–300 ms. Further repeated-measure ANOVAs were calculated to analyze ERP data with feedback valence (reward versus punishment) and feedback type (monetary versus social) as the within-subject factors and gender groups (boys versus girls) as the between-subject factor. Greenhouse–Geisser corrections were applied for the *p* values in all the ANOVAs. All *p* values less than 0.05 was considered statistically significant.

#### 2.6.3. FRN as a neural correlation of sensitivity to reward and punishment

Multiple regression analyses were performed to examine whether FRN component measures (FRN amplitude and FRN latency) could predict sensitivity to punishment and reward for boys and girls. For all the regression analyses, we controlled for the age of the children as relevant covariates by entering these data into the first block. The ERP measures, as continuous independent variables, were entered into the second block. To further secure unbiased regression models, FRN component measures for each feedback type (monetary or social) was entered in separate regression models. In particular, two sets of linear regression analyses were performed with the FRN elicited by monetary feedback (the FRN in the monetary reward and monetary punishment feedback, i.e., FRN MR and FRN MP), and consecutively, with the FRN elicited by social feedback (the FRN in the social reward feedback and social punishment feedback, i.e., FRN SR and FRN SP), as predictors. No alarming multicollinearity problem was detected with variance inflation factors ranging from 1.02 to 3.00, which were less than 10 across the models [40].

## 3. Results

### 3.1. Behavioral results

#### 3.1.1. The Sensitivity to Punishment and Sensitivity to Reward Questionnaires (SPSRQ)

In terms of individual differences in sensitivity to punishment and sensitivity to reward, no significant difference was found for the levels of sensitivity to punishment and sensitivity to reward between boys and girls (sensitivity to punishment: 36.58 (boys) versus 42.00 (girls), *t*(42) = −1.671, *p* > 0.05; sensitivity to reward: 47.79 (boys) versus 50.70 (girls), *t*(42) = −0.85, *p* > 0.05).

#### 3.1.2. Switching choice behavior

**Fig 3:**
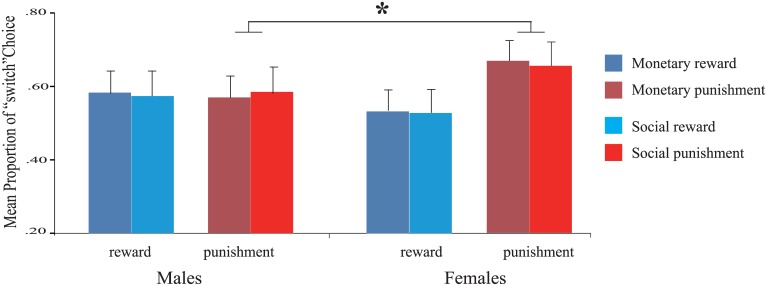
Switching choice behavior. Switching choice behavior following reward (blue bar) and punishment (red bar) feedback in the monetary (left) and social (right) tasks for boys and girls.

We first investigated whether the proportion of “switched” choices was influenced by the feedback condition obtained in the preceding trial. The participants were more likely to switch choice following punishment feedback (*M* = 0.62, SD = 0.14) than following reward feedback (*M* = 0.56, SD = 0.13; *F*(1, 42) = 5.44, *p* < 0.05, *η*^*2*^_partial_ = 0.115). This effect interacted significantly with gender (*F*(1, 42) = 4.94, *p* < 0.05, *η*^*2*^_partial_ = 0.105). As illustrated in Fig 3, a further simple effect test showed that boys (*M* = 0.58, SD = 0.15) were less likely to switch their choice following punishment than girls (*M* = 0.67, SD = 0.12; *F*(1, 42) = 5.20, *p* < 0.05). Neither significant main effect of other factors nor significant interaction between gender and other factors (all *p*s > 0.43) was found.

#### 3.1.3. Reaction time

The main effect was only significant for feedback type, with longer reaction time following monetary feedback (*M* = 1102.72 ms, SD = 351.98) than social feedback (*M* = 989.19 ms, SD = 326.27; *F*(1, 42) = 12.69, *p* < 0.05, *η*^*2*^_partial_ = 0.232). However, this effect exhibited no interaction with gender (*F*(1, 42) < 1) or feedback valence (*F*(1, 42) = 2.71, *p* > 0.05).

To summarize, both boys and girls were more likely to switch their choice after punishment than after reward, and girls performed significantly more switching responses than boys under the punishment condition. In addition, the participants demonstrated longer reaction time after monetary feedback than after social feedback. Although feedback was presented in random order, and thus, not related to the choices made, the behavior of the children indicated that they were sensitive to the outcomes of their choices, which was consistent with the result of a previous study [16].

### 3.2. ERP results

**Fig 4:**
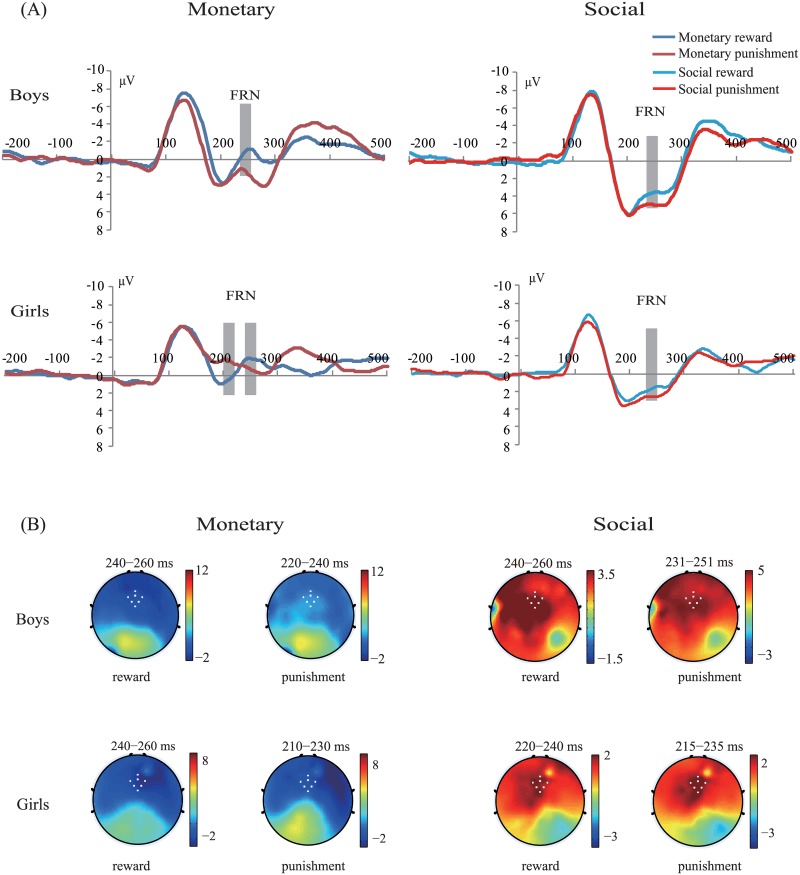
Grand average waves and topographic maps of FRNs. (A) Grand average ERPs that depict FRN in the reward (blue line) and punishment (red line) feedback in the monetary (left) and social (right) tasks for boys and girls. Gray bars highlight the time windows used for statistical analysis. (B) Topographic maps display the ERPs for reward and punishment feedback at the indicated time windows.

**Fig 5:**
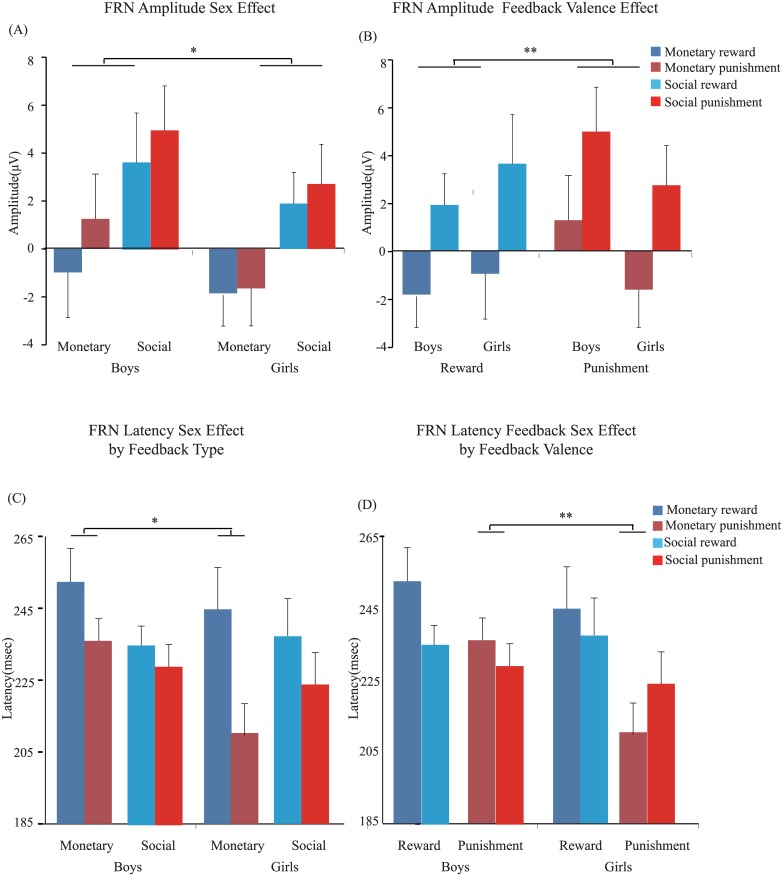
FRN peak amplitudes and latencies under all conditions (monetary reward, monetary punishment, social reward, and social punishment).

Previous works have suggested that FRN approximately occurs at 200–300 ms [9, 41] or at a later interval of 300 ms after feedback onset [14]. As illustrated in Fig 4, FRN occurs between 200–300 ms. For FRN amplitude, boys (*M* = 2.22 μV, SD = 5.03) presented less FRN peak amplitude than girls (*M* = 0.28 μV, SD = 3.67; the main effect of gender: *F*(1, 42) = 4.27, *p* < 0.05, *η*^*2*^_partial_ = 0.09; Figs 4 and 5A). In addition, the punishment feedback (*M* = 1.94 μV, SD = 4.58) elicited less negative peak amplitude than the reward feedback (*M* = 0.74 μV, SD = 4.47; main effect of feedback valence: *F*(1, 42) = 8.19, *p* < 0.01, *η*^*2*^_partial_ = 0.16; Figs 4 and 5B). No other effect, including triple interaction, was significant (all *p*s > 0.05).

We further analyzed FRN latency, and the results showed that boys (*M* = 238.08 ms, SD = 18.38) had a longer FRN latency than girls (*M* = 229 ms, SD = 24.58; the main effect of gender: *F*(1, 42) = 8.22, *p* < 0.05, *η*^*2*^_partial_ = 0.16; Figs 4 and 5C). This two-way interaction of gender effect by feedback type (*F*(1, 42) = 8.39, *p* < 0.05, *η*^*2*^_partial_ = 0.17; Figs 4 and 5C) and by feedback valence (*F*(1, 42) = 5.59, *p* < 0.05, *η*^*2*^_partial_ = 0.12; Figs 4 and 5D) was significant. However, their triple interaction did not achieve significance (*F*(1, 42) < 1). Further simple effect tests indicated that boys had a longer FRN latency than girls under the monetary condition (boys: *M* = 244.13 ms, SD = 20.30; girls: *M* = 227.50 ms, SD = 27.45; *F*(1, 42) = 15.93, *p* < 0.05) as well as under the punishment condition (boys: *M* = 232.50 ms, SD = 14.95; girls: *M* = 210.30 ms, SD = 17.19; *F*(1, 42) = 23.66, *p* < 0.05).

### 3.3. EEG predictors of sensitivity to reward and punishment

**Fig 6:**
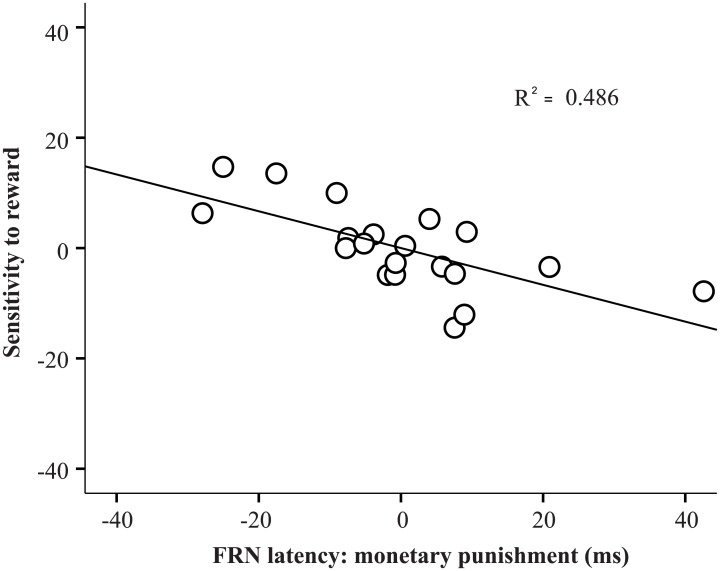
Scatterplot that illustrates the relationship between sensitivity to reward (*y*-axis, centered values) and FRN peak latency for monetary punishment signals (*x*-axis, centered values).

Two feedback types (monetary and social feedback) related to FRN component measures were entered into separate regression models for boys and girls (Tables 1 and 2). For sensitivity to reward, regression analyses regarding FRN in response to monetary feedback for girls showed that the linear combination of these ERP measures predicted scores for sensitivity to reward (after controlling for age, *R*^*2*^_change_ = 0.512, *F*(4, 13) = 3.964, *p* < 0.05). Only one significant effect was observed for the relationship between FRN latency in punishment condition and sensitivity to reward for girls (Fig 6). In particular, a longer FRN latency in monetary punishment predicted a lower score for sensitivity to reward for girls (*β* = −0.667, *p* < 0.005). However, no further ERP index prediction effect for boys and girls was found in the regression analysis regarding FRN in response to monetary/social feedback for sensitivity to reward.

**Table 1:** Regression results of FRN amplitude and latency measures in monetary feedback that predicts sensitivity to punishment and sensitivity to reward for girls and boys.

Variables	Boys				Girls			
	Reward Sensitivity		Punishment Sensitivity		Reward Sensitivity		Punishment Sensitivity	
	*β*	*ΔR*^*2*^	*β*	*ΔR*^*2*^	*β*	*ΔR*^*2*^	*β*	*ΔR*^*2*^
Step 1		0.044		0.020		0.074		0.005
Age	0.210		0.140		0.273		0.072	
Step 2		0.201		0.222		0.512*		0.060
FRN amplitude (MR)	0.062		0.068		0.092		0.261	
FRN latency (MR)	0.152		0.440		0.127		0.029	
FRN amplitude (MP)	0.370		0.038		0.157		0.154	
FRN latency (MP)	0.235		0.157		0.667***		0.037	

Note: MR = monetary reward, MP = monetary punishment

* *p* < 0.05,

**p < 0.01,

*** *p* < 0.005

**Table 2:** Regression results of FRN amplitude and latency measures in social feedback that predicts sensitivity to punishment and sensitivity to reward for girls and boys.

Variables	Boys				Girls			
	Reward Sensitivity		Punishment Sensitivity		Reward Sensitivity		Punishment Sensitivity	
	*β*	*ΔR*^*2*^	*Β*	*ΔR*^*2*^	*β*	*ΔR*^*2*^	*β*	*ΔR*^*2*^
Step 1		0.044		0.020		0.074		0.005
Age	−0.210		−0.140		−0.273		−0.072	
Step 2		0.101		0.188		0.033		0.132
FRN amplitude (SR)	0.068		0.077		0.177		−0.336	
FRN latency (SR)	0.021		0.353		0.028		0.275	
FRN amplitude (SP)	0.176		0.138		−0.117		0.003	
FRN latency (SP)	−0.219		−0.004		−0.148		−0.032	

Note: SR = social reward, SP = social punishment

For sensitivity to punishment, no ERP index prediction effect for both boys and girls was found in the regression analyses regarding FRN in response to monetary/social feedback (Tables 1 and 2).

## 4. Discussion

The current study examined gender differences in the neural responses to reward and punishment both in the contexts of monetary and social feedback during childhood. In addition, we further explored the relationship between the neural correlation of feedback processing and individual difference of sensitivity to reward and sensitivity to punishment for both boys and girls. The results showed that the FRN obtained in our study occurred between 200–300 ms and were maximal in the frontocentral regions, which were similar to those observed in considerable FRN literature about adults [9, 10, 16, 41, 42, 43, 44] and children [25, 45]. Our results showed that gender differences occurred in feedback processing. In particular, boys were less likely to switch their response after punishment, and had generally less FRN amplitude and longer FRN latency under monetary and punishment conditions than girls. In addition, FRN for monetary punishment, which was related to individual difference in reward sensitivity, was observed only in girls.

A debate on whether more negative FRN can be observed for the reward feedback than for the punishment feedback has recently started. Most prior research showed that FRN amplitude was more negative after punishment feedback than after reward feedback [25, 46]. However, two recent studies yielded opposite results. Flores et al. [20] found that reward feedback elicited more negative FRN than nonreward feedback in the case of monetary feedback. Stavropoulos and Carver [19] first utilized social versus nonsocial feedback. They also found that normally developing children aged 6 to 8 years had more negative FRN response to reward feedback than to punishment feedback when regarding social stimuli. In the current study, we observed that both boys and girls exhibited more negative FRN to reward feedback than to punishment feedback, regardless of feedback type. Therefore, how can these comparable negativities that occur after reward feedback be explained? FRN was initially assumed to reflect negative prediction errors [5]. However, recent reports of comparable negative FRNs following positive outcomes have led to new interpretations [20]. For instance, Holroyd et al. [47, 48] suggested that the FRN was actually an N2 component that was elicited by unexpected events irrespective of valence. This finding is also consistent with the evidence that surprise signals, rather reward prediction effect signals, modulate FRN amplitude and are directly projected onto the FRN source region [10]. However, in the present study, there is no solid reason to assume that reward is more unpredicted than punishment, because reward and punishment feedback randomly appeared with equal probability (50%). Therefore, further studies are needed to elucidate why children have more negative FRN to reward feedback than negative feedback when they were in an uncertain situation.

### 4.1. Gender difference in feedback processing

#### 4.1.1. Gender difference in reward and punishment feedback processing

The current study found that evidence for gender differences in feedback processing was provided by behavioral data, ERP data, and correlation data between ERP data and individual difference. First, gender difference in FRN amplitude was observed, irrespective of feedback valence and feedback type. In particular, boys generally exhibited more reduced FRN amplitude than girls. This result extended the findings of Santesso et al. [3], who reported that males had reduced FRN amplitude than females during adolescence and adulthood. As mentioned earlier, FRN does not only reflect a prediction error of the difference between the expected and actual outcomes [41, 49], but also affects feedback [3]. Santesso et al. [3] suggested that similar to error-related negativity, reduced FRN might be associated with hypovigilance or a lack of concern over the feedback of events. Moreover, this differential neural activity of feedback processing between boys and girls possibly has behavioral consequences. The behavior data of the current study found that compared with girls, boys were less likely to switch their response preceding a choice following punishment, which suggested that boys might not be concerned about feedback and might not exert additional effort to avoid punishment, which induced the reduced FRN. Thus, in the current study, boys had less FRN compared with girls in response to feedback, which suggested that they were less vigilant or concerned about external feedback.

As reviewed in the Introduction, findings regarding gender difference in feedback processing are conflicting. For example, Kujawa et al. [23] found that males had a larger ΔFN than females, whereas Bress et al. [24] found no ΔFN difference between the genders. One possible explanation for the discrepancy is that results differ between methods. For example, Banis and Lorist [16] measured FRN using three different approaches, and compared the findings among these three measures. They found that results differed among measures. Kujawa et al. [23] and Bress et al. [24] regarded the loss-minus-gain difference wave as FRN. By contrast, Crowley et al. [25] measured the mean amplitudes of original ERP waves as FRN. Therefore, one possible reason for the conflicting findings regarding gender difference in feedback processing may relate to different FRN measures.

Another gender difference observed in the current study is that the FRN latencies of boys and girls are contrarily affected by feedback valence and feedback type. In particular, boys demonstrated longer latency in the FRN for punishment than girls. Our result is consistent with that of Crowley et al. [25], who have investigated the feedback process for children and adolescents by adopting a reward versus nonreward task. They found that boys exhibited longer FRN latency than girls for reward, thereby suggesting gender difference in the neural architecture that supported FRN. Thus, the result of the current study suggests that boys are lagging behind girls in the internal representations of feedback ability. Moreover, compared with boys, girls have a tendency to obtain higher scores for punishment sensitivity (42.00 vs. 36.58). Girls who are highly sensitive to punishment may have accelerated FRN in response to punishment, particularly monetary punishment. Therefore, the finding suggests that gender difference in the latency of FRN may also explain gender difference in sensitivity to punishment.

#### 4.1.2. Gender difference in monetary and social feedback processing

With regard to feedback type, we found that the children exhibited longer reaction time after monetary feedback than after social feedback, thereby suggesting that children aged 9–12 years might particularly care about monetary feedback in general. This view was supported by the study of Kohls and Herpertz- Dahlmann [27], which found that larger effects were observed for monetary reward than for social reward, thereby suggesting that social feedback do not have an equally strong reinforcing value compared with monetary feedback for children aged 8–12 years. Moreover, as mentioned earlier, we found that boys had longer FRN latency under monetary condition than girls. This finding suggests a gender difference in the neural architecture that supports FRN. By contrast, no gender difference was found in the FRN for social feedback, which suggested that social feedback elicited similar arousal levels and processing patterns in girls and boys aged 9–12 years. Previous findings have emerged in the EEG and fMRI literature on social processing that suggest that females are more engaged in social tasks, however, most of these studies focused on adolescents and adults [50, 51, 52]. The aforementioned findings may account for the fact that gender difference in gonadal hormones, and possibly, the pubertal timing of social reward systems in the brain, including the detection of social–emotional stimuli, affects reactions to such stimuli and the regulation of these reactions through higher–order cognitive processes [53]. Therefore, future studies might be benefit from examining sex differences in the neural activity with social vs money feedback from childhood to adulthood.

### 4.2. FRN as a neural correlation of sensitivity to punishment

Consistent with our expectation, FRN in response to punishment was related to individual differences in reward sensitivity, which was moderated by gender. That is, our results showed that a long FRN latency for monetary punishment feedback was significantly associated with a low score on sensitivity to reward for girls, but not for boys. Torrubia et al. [54] indicated the two scales of the questionnaire (sensitivity to reward and sensitivity to punishment) were developed by writing items to assess BAS (Behavioural Approach System) and BIS (Behavioural Inhibition System) functioning, respectively. In line with the revised Reinforcement Sensitivity Theory [55], Corr [56] proposed that BAS antagonized the process of punishment stimuli, i.e. low-BAS individuals should demonstrate the highest aversive response and negative emotions to these stimuli. In the present study, a low sensitivity to reward was related to a long FRN latency. This relationship may be possibly explained by the point of view that individuals with low sensitivity to rewards require a long time to process punishment to digest the negative emotions. Moreover, such findings are only observed in girls, which may be due to sex-specific origin mechanisms that underpin the ERP component. As stated earlier, compared with boys, girls exhibit the tendency to obtain higher scores for punishment sensitivity. Clinical studies have found gender differences in the prevalence of depression [57, 58], which is linked to abnormal reward-related processing [59]. Although the role of gender on the relationship between FRN and psychometric risk for psychotic symptoms remains unclear, prior studies have found that the relationship between reduced frontal P300 amplitude and psychometric risk for psychotic symptoms is more evident among girls [60, 61]. However, we found that FRN latency in response to punishment was related to individual differences in reward sensitivity, but not in punishment sensitivity. Therefore, caution should be exercised in interpreting this relationship in the current study.

### 4.3. Conclusions

In conclusion, the current study showed that gender differences exist in the cognitive and neural performances of feedback processing during childhood. Compared with girls, boys were less likely to switch their response after punishment, and had less negative FRN amplitude and longer FRN latency. Furthermore, FRN in response to punishment related to individual differences in reward sensitivity was only observed in girls. This knowledge may provide educational guidance for providing appropriate feedback according to the developmental characteristics of children.

## Supporting information

S1 Data The minimal data set. (ZIP)
